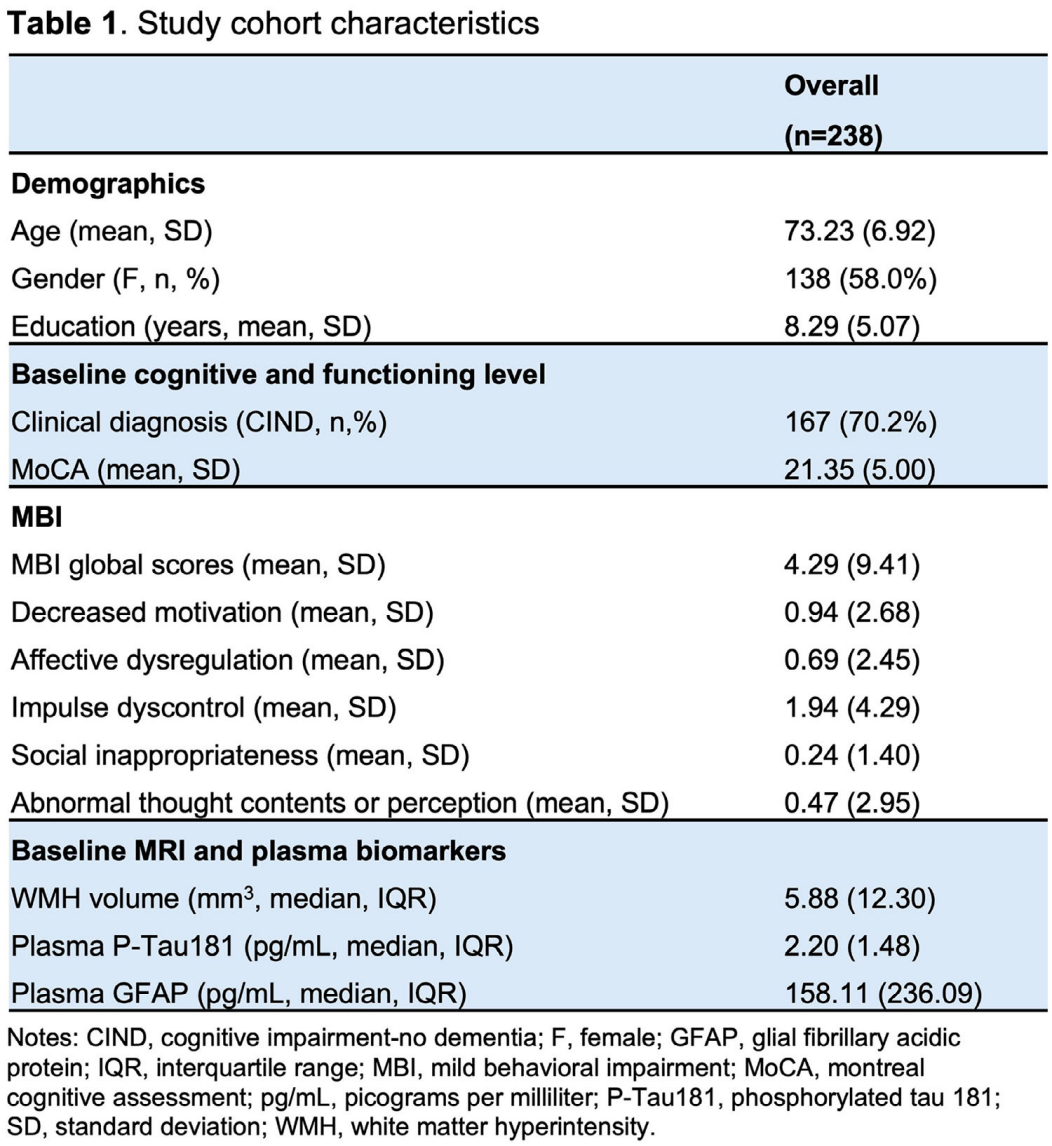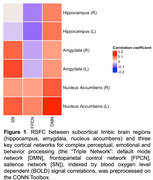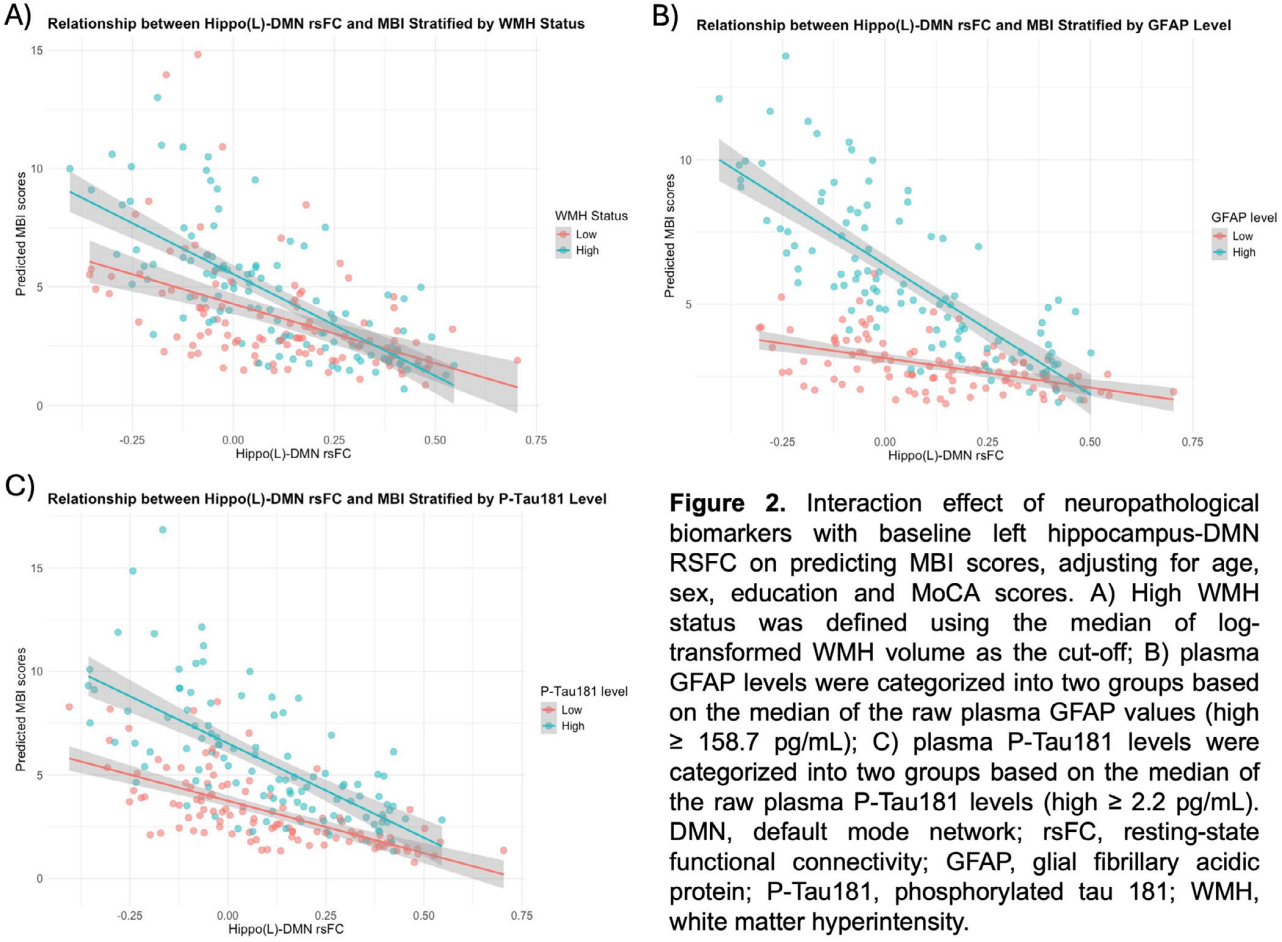# Cortical‐subcortical functional connectivity alterations are associated with mild behavioral impairment in Asian dementia‐free older adults

**DOI:** 10.1002/alz70856_101460

**Published:** 2025-12-24

**Authors:** Yingqi Liao, Cheuk Ni Kan, Shi Yu Chan, Joyce R Chong, Christopher Chen, Ai Peng Tan

**Affiliations:** ^1^ Memory, Ageing, and Cognition Centre (MACC), Department of Pharmacology, Yong Loo Lin School of Medicine, National University of Singapore, Singapore, Singapore; ^2^ Institute for Human Development and Potential (IHDP), Agency for Science, Technology and Research (A*STAR), Singapore, Singapore, Singapore; ^3^ Department of Psychological Medicine, Yong Loo Lin School of Medicine, National University of Singapore, Singapore, Singapore, Singapore; ^4^ Yong Loo Lin School of Medicine, National University of Singapore, Singapore, Singapore, Singapore; ^5^ Department of Diagnostic Imaging, National University Health System, Singapore, Singapore, Singapore

## Abstract

**Background:**

Mild behavioral impairment (MBI) is an early neuropsychiatric syndrome associated with an increased risk of dementia, yet its underlying mechanisms remain unclear. Altered functional connectivity (FC) between cortical networks including default mode network (DMN) and frontoparietal control network (FPCN) were observed in MBI. This current study aims to further examine the cortical‐subcortical FC correlates of MBI, and their relationship with Alzheimer's and cerebrovascular disease biomarkers.

**Method:**

Dementia‐free participants from the memory clinic (*n* = 238, age:73.2 ± 6.9, 138(58%) females, Table 1) underwent neuropsychological testing and resting‐state functional MRI brain scans at baseline. Global MBI and subdomain scores were indexed by summing the severity x frequency scores of Neuropsychiatric Inventory assessment across baseline and year 1 follow‐up. Resting‐state functional connectivity (RSFC) between subcortical limbic brain regions (i.e. hippocampus, amygdala and nucleus accumbens) and three key cortical networks for complex perceptual, emotional and behavior processing (i.e. DMN, FPCN and salience network (SN)) were generated (Figure 1). Zero‐inflated negative binomial regression was conducted to examine the association between MBI and cortical‐subcortical RSFC. RSFC measures that were significantly associated with MBI scores were then examined for their interaction with white matter hyperintensities volume (WMHV), plasma phosphorylated tau 181 (*p*‐Tau181) and glial fibrillary acidic protein (GFAP), in relation to MBI scores. All models were adjusted for age, sex, education and MoCA scores. The false discovery rate approach (*p* <0.05) was employed to correct for multiple comparisons.

**Result:**

Reduced left hippocampus‐DMN RSFC was associated with greater MBI global scores (𝜷[95%CI]=‐1.54[‐2.39,‐0.69]), driven by decreased motivation (𝜷[95%CI]=‐1.25[‐2.13,‐0.38]) and affective dysregulation (𝜷[95%CI]=‐2.24[‐3.83,‐0.65]) subdomains. The association between left hippocampus‐DMN RSFC and MBI global scores was significantly moderated by WMHV (𝜷[95%CI]=‐0.83[‐1.57,‐0.08]) and showed a marginally significant moderation by GFAP (𝜷[95%CI]=‐0.74[‐1.51,0.03]). In contrast, no significant moderation by *p*‐Tau181 was observed (*p* = 0.689). Notably, the significant association between left hippocampus‐DMN RSFC and MBI global scores was only present when there was high WMHV or high GFAP (Figure 2).

**Conclusion:**

Our findings demonstrated the association between cortical‐subcortical functional alterations in DMN and MBI. Furthermore, cerebrovascular pathology and neuroinflammation processes may play an important role in modulating this brain‐behavior relationship, which warrants for further investigation.